# Identification of *Pappa* and *Sall3* as Gli3 direct target genes acting downstream of cilia signaling in corticogenesis

**DOI:** 10.1093/cercor/bhae480

**Published:** 2024-12-24

**Authors:** Shinjini Basu, Lena Mautner, Kae Whiting, Kerstin Hasenpusch-Theil, Malgorzata Borkowska, Thomas Theil

**Affiliations:** Centre for Discovery Brain Sciences, University of Edinburgh, Edinburgh, United Kingdom; Simons Initiative for the Developing Brain, University of Edinburgh Hugh Robson Building, Edinburgh, United Kingdom; Centre for Discovery Brain Sciences, University of Edinburgh, Edinburgh, United Kingdom; Centre for Discovery Brain Sciences, University of Edinburgh, Edinburgh, United Kingdom; Centre for Discovery Brain Sciences, University of Edinburgh, Edinburgh, United Kingdom; Simons Initiative for the Developing Brain, University of Edinburgh Hugh Robson Building, Edinburgh, United Kingdom; Centre for Discovery Brain Sciences, University of Edinburgh, Edinburgh, United Kingdom; Centre for Discovery Brain Sciences, University of Edinburgh, Edinburgh, United Kingdom; Simons Initiative for the Developing Brain, University of Edinburgh Hugh Robson Building, Edinburgh, United Kingdom

**Keywords:** corticogenesis, *Gli3*, *Inpp5e*, *Pappa*, primary cilia, *Sall3*

## Abstract

The cerebral cortex is critical for advanced cognitive functions and relies on a vast network of neurons to carry out its highly intricate neural tasks. Generating cortical neurons in accurate numbers hinges on cell signaling orchestrated by primary cilia to coordinate the proliferation and differentiation of cortical stem cells. While recent research has shed light on multiple ciliary roles in corticogenesis, specific mechanisms downstream of cilia signaling remain largely unexplored. We previously showed that an excess of early-born cortical neurons in mice mutant for the ciliary gene *Inpp5e* was rescued by re-introducing Gli3 repressor. By comparing expression profiles between *Inpp5e* and *Gli3* mutants, we here identified novel Gli3 target genes. This approach highlighted the transcription factor gene *Sall3* and *Pappalysin1* (*Pappa*), a metalloproteinase involved in IGF signaling, as upregulated genes in both mutants. Further examination revealed that Gli3 directly binds to *Sall3* and *Pappa* enhancers and suppresses their activity in the dorsal telencephalon. Collectively, our analyses provide important mechanistic insights into how primary cilia govern the behavior of neural stem cells, ultimately ensuring the production of adequate numbers of neurons during corticogenesis.

## Introduction

The cerebral cortex consists of dozens of different types of neurons to perform highly complex neural tasks (van den Ameele et al. 2014). Understanding how these neurons are generated in correct quantities, at the right time and place poses a major challenge. Corticogenesis entails a multistep process beginning with the subdivision of the telencephalon into distinct dorsal and ventral domains that give rise to the neocortex and the basal ganglia, respectively. This patterning process coincides with an expansion of cortical stem and progenitor cells that eventually undergo neurogenesis to form the various neuronal subtypes in a coordinated manner. These processes heavily rely on extensive cell signaling facilitated by primary cilia, tiny cell surface protrusions that act as antennae for cell signals. Cilia are critically important for controlling cortical growth in mice (Wilson et al. 2012; Foerster et al. 2017) and in humans (Budny et al. 2006; Davis et al. 2007; Putoux et al. 2011; Bachmann-Gagescu et al. 2012; Jamsheer et al. 2012) and regulate the activity of signaling pathways essential for cortical progenitor development (Wilson et al. 2012; Foerster et al. 2017). Notably, they govern the formation of the Gli3 repressor (Gli3R) crucial for cortical growth (Wang et al. 2011, 2014; Wilson et al. 2012; Hasenpusch et al. 2018, 2020). These findings strongly support a role for cilia in controlling cortical stem cell behavior, but the underlying mechanisms have hardly been investigated.

We recently addressed this question by analyzing mice with a mutation in the *Inositol Polyphosphate-5-Phosphatase E* (*Inpp5e*) gene which regulates the phosphoinositol composition of the cilium and thereby ciliary protein trafficking and signaling (Chavez et al. 2015; Garcia-Gonzalo et al. 2015; Constable et al. 2020; Hasenpusch et al. 2020). The analysis of *Inpp5e* mutants unveiled a profound role of cilia in cortical stem cells since mutant radial glial cells (RGCs) predominately underwent direct neurogenesis resulting in increased deep layer neuron formation (Hasenpusch et al. 2020). This phenotype coincided with reduced Gli3R formation and was remarkably rescued by re-introducing Gli3R. Additionally, human cortical organoids lacking *INPP5E* function were ventralized due to reduced GLI3R levels and increased SHH signaling (Schembs et al. 2022). These findings indicate an evolutionarily conserved role of *INPP5E* in controlling GLI3R formation during corticogenesis but the downstream genes and processes remained unclear.

Here, we systematically analyzed cortical development in *Inpp5e* mutant mice using gene expression profiling. A comparison with an mRNA-seq data set from *Gli3* conditional mouse mutants (Hasenpusch et al. 2018) revealed a significant overlap in differentially expressed genes (DEGs) suggesting a convergence onto a common phenotype. As Gli3 primarily acts as a transcriptional repressor during corticogenesis (Fotaki et al. 2006), we focussed on a common set of upregulated genes involved in dorsal/ventral patterning, cilia disassembly and known Sonic hedgehog target genes. *Pappalysin1* (*Pappa*), a regulator of insulin growth factor (IGF) signaling (Lawrence et al. 1999), and the transcription factor gene *Spalt-like 3* (*Sall3*) were among the most strongly upregulated genes and were ectopically expressed in the mutant dorsal telencephalon. Furthermore, Gli3 protein bound to *Pappa* and *Sall3* enhancers, and mutations in these Gli3 binding sites led to ectopic enhancer activity in cortical progenitors. These findings establish *Pappa* and *Sall3* as novel Gli3 target genes and suggest their involvement downstream of cilia signaling and Gli3R in controlling cortical neurogenesis.

## Material and methods

### Mice

All experimental work was carried out in accordance with the UK Animals (Scientific Procedures) Act 1986 and UK Home Office guidelines. All protocols were reviewed and approved by the named veterinary surgeons of the College of Medicine and Veterinary Medicine, the University of Edinburgh, prior to the commencement of experimental work. *Inpp5e*^Δ/+^, *Gli3* conditional (*Gli3*^fl^), and *Gli3*^Δ699/+^ mouse mutants and the *Emx1Cre* driver line have been described previously (Böse et al. 2002; Gorski et al. 2002; Blaess et al. 2008; Jacoby et al. 2009). *Inpp5e*^Δ/+^ mice were interbred to generate *Inpp5e*^Δ/Δ^ embryos; exencephalic *Inpp5e*^Δ/Δ^ embryos which made up ca. 25% of homozygous mutant embryos were excluded from the analyses. Wild-type and *Inpp5e*^Δ/+^ litter mate embryos served as controls. *Inpp5e*^Δ/Δ^;*Gli3*^Δ699/+^ embryos were obtained from crosses of *Inpp5e*^Δ/+^;*Gli3*^Δ699/+^, and *Inpp5e*^Δ/+^ mice using wild-type, *Inpp5e*^Δ/+^ and *Gli3*^Δ699/+^ embryos as controls. To generate *Gli3* conditional mutants, *Emx1Cre*;*Gli3*^fl/+^ mice were interbred with *Gli3*^fl/+^ animals; *Gli3^flox/flox^*, *Gli3^flox/+^,Emx1Cre* and *Gli3^flox/+^* embryos served as controls. Embryonic (E) day 0.5 was assumed to start at midday of the day of vaginal plug discovery. Transgenic animals and embryos from both sexes were genotyped as described (Jacoby et al. 2009; Hasenpusch-Theil et al. 2012). For each marker and each stage, 3–8 embryos were analyzed.

### In situ hybridization, immunohistochemistry, and X-Gal staining on sectioned embryonic brains

In situ hybridization on 12 μm coronal paraffin sections of E12.5 mouse brains were performed as described previously (Theil 2005). Digoxigenin-labeled antisense probes were generated from the following cDNA clones: *Pappa* (Genepaint riboprobe T37932) and *Sall3* (Genepaint riboprobe T38908).

For the reporter gene analysis of in utero electroporated embryos, brains were dissected in Phosphate Buffered saline (PBS) and fixed for 3 h in 4% paraformaldehyde (PFA). After embedding in Optimal Cutting Temperature (OCT) embedding medium/sucrose, 14 μm coronal cryosections were analyzed by immunofluorescence using a polyclonal chicken antibody against GFP (1:1,000; Abcam ab13970), followed by a nuclear counterstain with TO-PRO-1 (1:2,000, Invitrogen) as described previously (Hasenpusch-Theil et al. 2017). Adjacent sections were stained between 3 and 24 h with X-Gal at 37°C and counterstained with Fast RED (Hasenpusch-Theil et al. 2017).

### Plasmid construction and mutagenesis

All genomic DNA fragments were generated via PCR using wild-type genomic DNA (for oligonucleotides, see Table S2). Enhancer sequences were subcloned using a TOPO TA cloning kit (Invitrogen) and verified by sequencing. Putative Gli3 binding sites were mutated using the QuickChange Site-Directed Mutagenesis Kit (Stratagene) (for oligonucleotides used in mutagenesis, see Table S3). All mutations were confirmed by sequencing. To test for enhancer activity, wild-type and mutant regulatory elements were subcloned into the *lacZ* reporter gene vector pGZ40 upstream of a human β*-globin* minimal promoter (Yee and Rigby 1993).

### Electrophoretic mobility shift assay

Electrophoretic mobility assays were performed with biotin labeled oligonucleotides using purified GST and GST-Gli3 fusion protein as described previously (Hasenpusch-Theil et al. 2017). The binding reactions were separated on native 5% acrylamide gels and transferred onto positively charged nylon membranes (Roche) with a Perfect Blue Semi-dry electro blotter (60 min at 120 V, 5 mA). After UV crosslinking, biotin labeled probes were detected using a Chemiluminescent Nucleic Acid Detection Module (Thermo Scientific #89880) according to manufacturer’s instructions and imaged using a Kodak BioMaxXAR film.

For oligonucleotide sequences covering the wild-type or mutated Gli3 binding sites, see Table S4. The exchanged nucleotides in the mutated forms are underlined. Wild-type and Gli3 binding site mutant oligonucleotides were used as specific and unspecific competitors, respectively, in a 10- to 100-fold molar excess.

### In utero electroporation

E13.5 pregnant mice were anesthetized with isoflurane and the uterine horns were exposed. *LacZ* reporter gene plasmids and a *GFP* expression plasmid were co-injected into the lateral ventricle at 1 mg/ml each with a glass micropipette. The embryo in the uterus was placed between CUY650 tweezer-type electrodes (Nepagene). A CUY21E electroporator (Nepagene) was used to deliver 6 pulses (30 V, 50 ms each) at intervals of 950 ms. The uterine horns were placed back into the abdominal cavity and embryos were allowed to develop for 24 h before further processing for immunofluorescence. For each construct, at least 3 different embryos were analyzed.

### Western blot

Protein was extracted from the dorsal telencephalon of E12.5 wild-type and *Inpp5e*^Δ/Δ^ embryos (*n* = 4 samples per genotype) as described previously (Magnani et al. 2010); 30 μg protein lysates were loaded on 4%–12% NuPAGE® Bis-Tris gels (Life Technologies) and later transferred to an Immobilon–FL membrane. Membranes were incubated with the following primary antibodies: rabbit anti-phospho-Akt^S473^ (1:1,000, Cell Signaling Technologies CST #9271) and rabbit anti-Akt (1:1,000, CST #9272). For the detection of phosphorylated proteins goat anti-rabbit IRDye 680RD (1:15,000, LI-COR Biosciences) and for total proteins goat anti-rabbit IRDye 800CW (1:15,000, LI-COR Biosciences) were used as secondary antibodies. After detecting the phospho-Akt^S473^ signal the blot was stripped using Re-Blot Plus Strong Solution (Millipore, #2504) according to the manufacturer’s instruction. The signals were detected via the Odyssey Imaging System and further analyzed using Image Studio Lite Version4.0. The ratios between phosphorylated and total protein were compared between wild-type and mutant tissue using a paired t-test.

### Bulk mRNA-seq and bioinformatic analyses

For bulk mRNA-seq experiments, dorsal telencephalic tissue was dissected from E12.5 *Inpp5e* mutant embryos to generate 4 different replicates per genotype (control: *Inpp5e*^+/+^ and *Inpp5e*^Δ/+^; mutant: *Inpp5e*^Δ/Δ^). Total RNA was extracted using RNeasy Plus Mini Kit (Qiagen). After assessing the integrity of the RNA samples with an Agilent 2100 Bioanalyzer (RIN > 8), all RNAs were further processed for RNA library preparation and sequenced (paired-end, 50 bp reads) on an Illumina NovaSeq platform at Edinburgh Genomics (University of Edinburgh). Sequencing quality was checked using FastQC software and reads were aligned to the *Mus musculus* reference genome (genome assembly mm10) and analyzed using STAR alignment software. The featureCounts tool (Liao et al. 2014) was used to quantify gene expression. Count normalization and differential gene expression analyses were conducted in RStudio using the DESeq2 package (Love et al. 2014). Principal component analyses and hierarchical clustering were applied to normalized count data. Genes were annotated the biomaRt software package (Durinck et al. 2009). DEGs were selected based on an adjusted *P*-value < 0.05 and are summarized in Table S1. Gene ontology (GO) analyses was performed using Clusterprofiler Software (Wu et al. 2021) in the annotation category BP. Strongly enriched terms had a score of < 0.05 after Benjamini–Hochberg multiple test correction.

## Results

### Gene expression profiling of *Inpp5e* mutant dorsal telencephalon

**Fig. 1:**
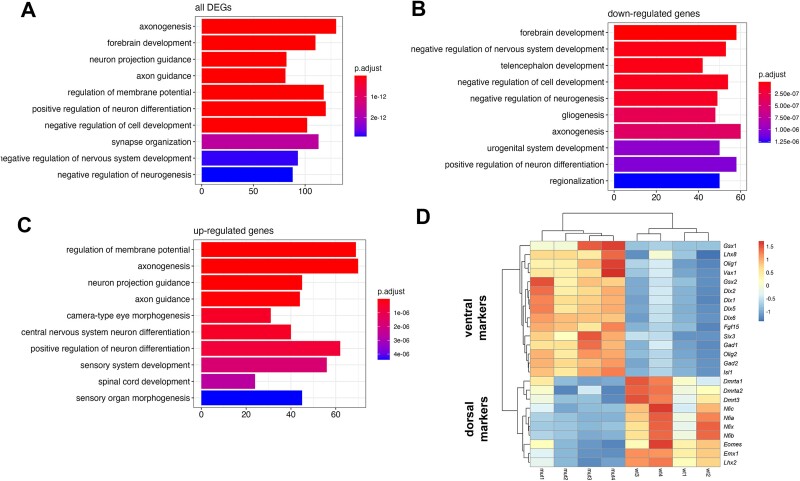
Differential gene expression in the E12.5 *Inpp5e*^Δ/Δ^ dorsal telencephalon. (A–C) GO analysis of all DEGs (A), and of only downregulated (B) or upregulated genes (C). (D) Heatmap comparing the expression of dorsal and ventral telencephalic markers.

We recently reported that the *Inpp5e* mutation alters the balance between direct and indirect neurogenesis (Hasenpusch et al. 2020). To explore broader gene expression changes underlying this phenotype, we performed bulk mRNA sequencing (mRNA-seq) to compare the expression profiles in the dorsal telencephalon of E12.5 control and *Inpp5e* mutant embryos. This analysis identified 2533 DEGs (padj < 0.05), with 1,329 upregulated and 1,204 downregulated genes (Table S1). GO analysis showed that these genes were primarily involved in neuronal differentiation (GO:BP terms: “regulation of neuron differentiation,” “axonogenesis,” “synapse organization,” “regulation of membrane potential”) and forebrain development (Fig. 1A). Downregulated genes related to “forebrain development” and “negative regulation of neurogenesis,” whereas upregulated genes were associated with “positive regulation of neurogenesis,” “axon guidance,” and “regulation of membrane potential” (Fig. 1B and C). These categorizations aligned with our previous observations of mild patterning, neurogenesis, and axon pathfinding defects in *Inpp5e* mutants (Magnani et al. 2015; Hasenpusch et al. 2020). To better understand how *Inpp5e* regulates these processes, we created a network plot of gene connections, highlighting *Fgfr3*, *Hairy and enhancer of split 1* (*Hes1*), and *Inhibitor of DNA binding 4* (*Id4*) among the downregulated genes (Fig. S1). These genes are involved in Fgf, Notch, and Bmp signaling, respectively, suggesting alterations in these pathways may contribute to the partial ventralization and increased neurogenesis. Accordingly, we observed upregulation of key regulators of ventral telencephalon development and downregulation of genes governing dorsal telencephalon development (Fig. 1D). Taken together, these findings support *Inpp5e*’s previously described roles in forebrain patterning and neuronal differentiation.

### Identification of genes acting downstream of *Gli3* in *Inpp5e* mutants

**Fig. 2:**
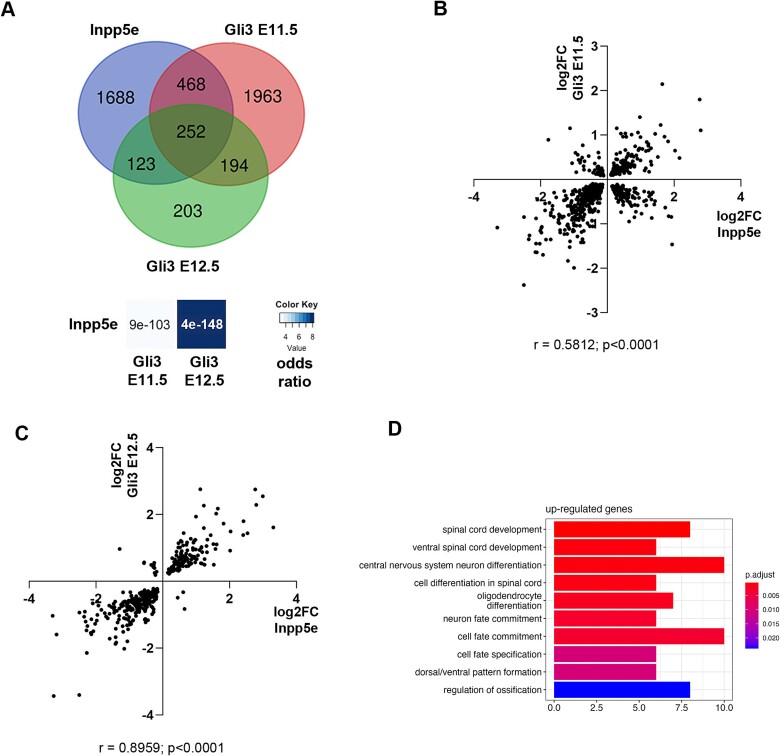
Comparison of differential gene expression between *Inpp5e* and *Gli3* mutants. (A) Venn diagram intersection of DEGS in E12.5 *Inpp5e*^Δ/Δ^, E11.5 and E12.5 *Gli3*^cKO^ embryos. Significance and odds ratio are indicated. (B, C) Comparison of gene expression changes between *Inpp5e*^Δ/Δ^ and E11.5 (B) and E12.5 (C) *Gli3*^cKO^ mutants. (D) GO analysis of genes upregulated in both mutants. Statistical tests: Fisher’s exact test (A) and Spearman correlation (B, C).

We previously reported that re-introducing Gli3R in *Inpp5e* mutants rescued the imbalance between direct and indirect neurogenesis (Hasenpusch et al. 2020). To identify downstream genes of *Gli3* that potentially mediate this rescue, we compared the *Inpp5e* gene expression profiling with our bulk mRNAseq analyses of dorsomedial telencephalon of E11.5 and E12.5 *Gli3* conditional mouse mutants in which *Gli3* is inactivated using an *Emx1Cre* driver line (Hasenpusch et al. 2018). Genes differentially expressed in both mutants are candidates to be regulated by *Gli3* in *Inpp5e* mutants. This comparison revealed statistically significant overlaps in DEGs between *Inpp5e* and *Gli3* mutants at E11.5 and E12.5 (Fig. 2A; Table S1) with nearly 50% of all DEGs in E12.5 *Gli3* mutants differentially expressed in *Inpp5e* embryos. We also observed correlations between the fold changes in the 2 mutants (Fig. 2B and C). While 24% of genes were regulated oppositely at E11.5, a remarkable 95% of DEGs were either upregulated or downregulated at E12.5 suggesting that both mutations converge on similar phenotypes.

As *Gli3* predominately acts as a repressor in cortical development (Fotaki et al. 2006), we focused on the 135 genes that were upregulated in both mutants at E12.5. Among the top 6 upregulated genes are *Gsx2*, *Olig1/2*, and *Fgf15*, critical for ventral telencephalic development. We also noted an increased expression of the Shh target genes *Patched1* (*Ptch1*) and *Cyclin D1* (*Ccnd1*), emphasized by GO:BP terms, such as “Dorsal/ventral pattern formation” and “oligodendrocyte differentiation” (Fig. 2D). Closer inspection also revealed an upregulation of *Histone deacetylase 6* (*Hdac6*), a regulator of ciliary disassembly (Pugacheva et al. 2007; Loktev et al. 2008; Forcioli-Conti et al. 2016; Lysyganicz et al. 2021), suggesting a novel feedback mechanism whereby cilia mediated Shh signaling stimulates *Hdac6* expression which in turn may lead to more labile or shorter cilia with reduced signaling capacity (Macarelli et al. 2023). Overall, these gene expression changes align with the partial ventralization of the dorsal telencephalon in both mutants and the destabilized cilia in *Inpp5e* mutant embryos.

### *Pappa* and *Sall3* expression are elevated in *Gli3* conditional and *Inpp5e* mutants

**Fig. 3:**
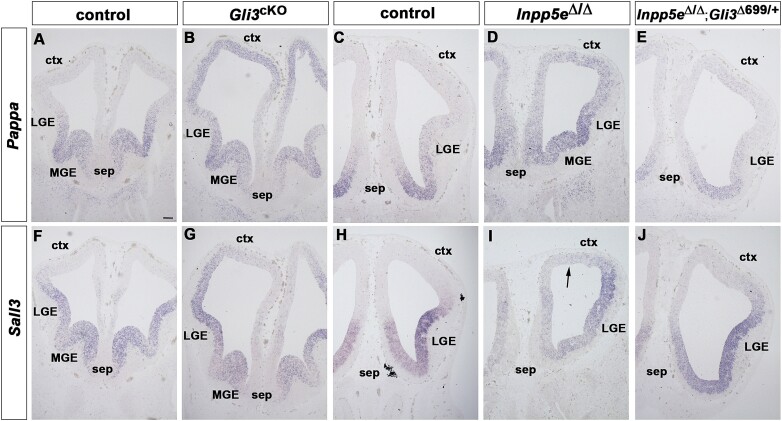
*Pappa* and *Sall3* expression in the *Gli3*^cKO^ and *Inpp5e*^Δ/Δ^ mutant telencephalon. Coronal sections of the E12.5 forebrain of the indicated genotypes were in situ hybridized with the indicated probes. (A, C) *Pappa* expression is confined to progenitors in the septum (sep), medial ganglionic eminence (MGE) and the ventral lateral ganglionic eminence (LGE) of control embryos. (B, D) Ectopic *Pappa* expression throughout the cortex (ctx) of *Gli3*^cKO^ embryos and in the dorsolateral telencephalon of *Inpp5e* mutant embryos. (F, H) *Sall3* expression is confined to the ventral telencephalon in control embryos. (G, I) *Gli3*^cKO^ embryos display ectopic *Sall3* transcripts in the developing cortex while *Inpp5e* mutants present with a more restricted up-regulation in the dorsal telencephalon (arrow). (E, J) *Pappa* and *Sall3* expression remain confined to the ventral telencephalon in *Inpp5e*^Δ/Δ^; *Gli3*^Δ699/+^ embryos. Scale bar: 200 mm.

The strong overlap of DEGs in *Inpp5e* and *Gli3* mutants provided us with a unique foundation for identifying genes that are controlled by ciliary signaling and act downstream of Gli3. To choose candidates for further analysis, we focussed on genes with known roles in embryonic pattern formation and/or cell signaling but previously not implicated in telencephalon development. Notably, *Sall3* and *Pappa* were among the most strongly upregulated genes and encode a zinc finger transcription factor and a zinc metalloproteinase involved in IGF signaling (Lawrence et al. 1999), respectively. In situ hybridization analysis revealed that in control embryos *Pappa* and *Sall3* transcripts were confined to ventral telencephalic progenitors with low *Pappa* gene expression levels in the dorsal lateral ganglionic eminence (Fig. 3A, C, F, H). In contrast, both genes were found to be ectopically expressed in the rostral cortex of both mutants but ectopic expression was less pronounced in *Inpp5e* mutants (Fig. 3B, D, G, I). These patterns confirmed the upregulation of *Pappa* and *Sall3* and validated our bulk mRNA-seq results.

### Gli3 binds to *Pappa* and *Sall3* forebrain enhancers in vivo and in vitro

**Fig. 4:**
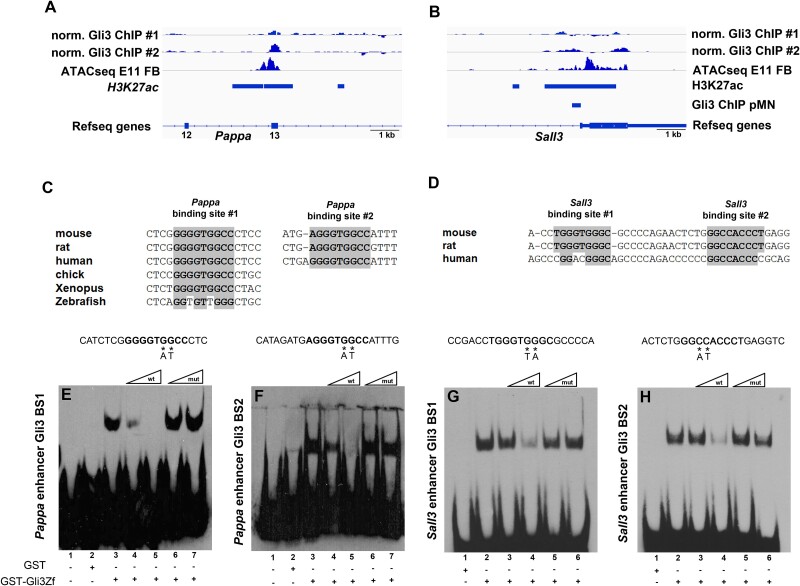
Gli3 binds to *Pappa* and *Sall3* enhancers in vivo and in vitro. (A, B) Genome browser snapshots showing Gli3 ChIP-peaks at exon 13 of *Pappa* overlapping with an open chromatin region (ATACseq peak) (A) and in the first intron of *Sall3* (B). The latter coincides with an H3K27ac positive region and a Gli3 ChIP-peak identified in motor neuron progenitors. (C, D) Evolutionary conservation of *Gli3* binding sites in the *Pappa* (B) and *Sall3* (D) enhancers. (E-H) Ectromobility shift assays demonstrating specific binding of a GST-Gli3 fusion protein to binding sites 1 (E) and 2 (F) of the *Pappa* enhancer and to sites 1 (G) and 2 (H) of the *Sall3* enhancer.

The upregulation of *Pappa* and *Sall3* suggested that Gli3 may directly control their expression by binding to and repressing gene regulatory elements in cortical cells, thereby restricting their transcription to the ventral telencephalon. This hypothesis is supported by the rescue of the *Pappa* and *Sall3* expression patterns in the telencephalon of *Inpp5e*^Δ/Δ^; *Gli3*^Δ699/+^ double mutants (Fig. 3E and J) in which the Gli3 repressor is formed in a cilia independent manner (Böse et al. 2002, 2011). To gain further evidence for a direct regulatory interaction, we next examined a Gli3 ChIP-seq data set (Hasenpusch et al. 2018) and identified a Gli3 peak within the *Pappa* gene overlapping with exon 13 and coinciding with a region of open chromatin in E11.5 forebrain tissue (ENCODE accession number ENCFF426VDN) (Fig. 4A). A 1 kb sequence surrounding exon 13 contained 3 potential *Gli3* binding sites. Site 1 located within exon 13 and showed high evolutionary conservation across several vertebrate species, whereas sites 2 and 3 within intron 12 were less conserved (Fig. 4C). Importantly, the human site 3 contained an A/T exchange in a critical nucleotide of the *Gli3* binding sequence and a GLI3 Cut&Tag experiment on human cortical organoids showed a GLI3 peak only encompassing sites 1 and 2, but not site 3 (Fleck et al. 2022). Hence, we focused our further analyses on sites 1 and 2.

For *Sall3*, we noted several Gli3 peaks surrounding the transcriptional start site (Fig. 4B). The intronic peak overlapped with an open chromatin region and with Gli3 binding peaks in murine motor neuron progenitors (Nishi et al. 2015) and human cortical organoids (Fleck et al. 2022). This region contained 2 adjacent *Gli3* binding sites in mouse and rat, whereas only 1 site was present in the human genome (Fig. 4D).

To confirm Gli3 binding to these sites in vitro, we utilized a GST-Gli3 fusion protein containing the Gli3 DNA binding domain in electromobility shift assays with biotin-labeled oligonucleotides encompassing the binding motifs from the *Pappa* and the *Sall3* genes. This approach resulted in the formation of a slower migrating complex for all binding sites (Fig. 4E–H). Competition assays using unlabeled wild-type oligonucleotide progressively reduced binding with increasing amounts of the competitor, whereas oligonucleotides with a GG to AT exchange, abolishing Gli binding (Hepker et al. 1999), did not affect complex formation. Thus, Gli3 specifically bound to sequences within the *Pappa* and *Sall3* genes.

### Gli3 represses *Pappa* and *Sall3* forebrain enhancer activity

**Fig. 5:**
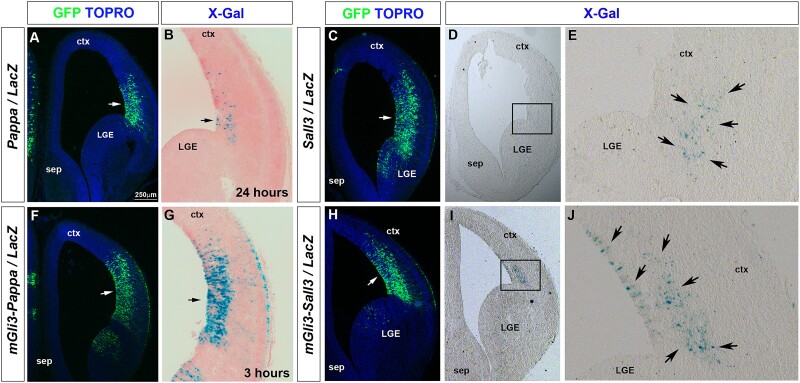
Gli3 represses activity of *Pappa* and *Sall3* enhancers in the dorsolateral telencephalon. Coronal forebrain sections of E14.5 embryos in utero electroporated with the indicated constructs were stained either with GFP antibodies or with X-Gal. GFP staining indicates the electroporated regions (arrows in A, C, F and H). (A-E) The *Pappa* and *Sall3* enhancers showed weak activity in a limited number of cells in the dorsolateral telencephalon. (F, G) Mutations in the *Gli3* binding sites led to strong reporter gene expression (arrow in G). Note the different staining times. (H-J) Activity of a *Gli3* binding site mutant *Sall3* enhancer was stronger and more widespread (arrows in J). Abbreviations: Ctx, cortex; MGE, medial ganglionic eminence; LGE, lateral ganglionic eminence; sep, septum. Scale bar: 250 mm.

Finally, we assessed the in vivo functionality of the Gli3 binding sites. We subcloned wild-type or *Gli3* binding motif mutant *Pappa* and *Sall3* enhancers into the pGZ40 reporter vector containing a *lacZ* reporter gene under the control of a human *beta-globin* minimal promoter. These reporter gene constructs were co-electroporated with a GFP expression plasmid into the forebrain of E13.5 embryos which were harvested 24 h post electroporation. Adjacent cryosections were subsequently stained with X-Gal and a GFP antibody to monitor enhancer activity and reveal transfected cells, respectively (Fig. 5). Despite extensive electroporation, the wild-type *Pappa* enhancer only exhibited mild activity in the dorsolateral telencephalon after 24 h of staining (Fig. 5A and B), consistent with *Pappa* gene expression being confined to the ventral telencephalon. In contrast, the mutant enhancer constructs elicited strong enhancer activity in dorsolateral cortical stem cells only after 3 h of staining (Fig. 5F and G). Similarly, the wild-type *Sall3* enhancer led to weak β-galactosidase staining in very few cells immediately dorsal to the pallial-subpallial boundary in 3 out of 5 electroporated brains (Fig. 5C–E). Embryos electroporated with the mutant *Sall3* enhancer showed many, strongly stained cells in an extended region in 3 out of 4 embryos (Fig. 5H–J). These findings suggest that the *Gli3* binding sites are essential elements in repressing *Pappa* and *Sall3* expression in the dorsal telencephalon.

## Discussion

Creating a fully functional cerebral cortex heavily relies on precise cell–cell communication facilitated by primary cilia. Our previous research has demonstrated a crucial ciliary role in regulating Gli3R levels to maintain the balance between direct and indirect neurogenesis but the exact downstream effectors of cilia and Gli3R remained elusive. In this study, we conducted a comparative analysis of differential gene expression in the developing cortex of *Gli3* mutants and of embryos mutant for the ciliary gene *Inpp5e*. This comparison revealed an overlap of 375 DEGs involved in key processes, such as D/V patterning, cell signaling, and neurogenesis. We further focussed our analyses on 2 specific genes, namely *Sall3* and *Pappa*, showing their direct regulation by Gli3. Taken together, these findings shed light on the mechanisms by which cilia orchestrate specific aspects of cortical development.

### Identification of ciliary and Gli3 target genes

Recently, significant progress has been made in understanding of how primary cilia control various signaling pathways crucial for corticogenesis. Most notably, cilia are essential for producing the Gli3 repressor which is critical not only for suppressing Sonic hedgehog signaling to prevent a ventralization of the developing cortex (Tole et al. 2000; Kuschel et al. 2003) but also for controlling the timing of neuronal differentiation in a Shh independent manner (Hasenpusch et al. 2018). Rescue experiments involving the reintroduction of Gli3R have underscored this important function and achieved remarkable recoveries in restoring cortical neurogenesis (Hasenpusch et al. 2020), olfactory bulb formation (Besse et al. 2011), and corpus callosum development (Laclef et al. 2015; Putoux et al. 2019) in several cilia mouse mutants. The genes, however, that act downstream of cilia and *Gli3* remain largely unidentified, but such knowledge is crucial for a comprehensive apprehension of ciliary functions.

To address this gap, we performed gene expression profiling of the *Inpp5e* mutant cortex and compared the DEGs with those from a similar experiment involving *Gli3* conditional inactivation in the cortex. This comparison revealed an overlap of 375 DEGs, accounting for nearly 50% of all DEGs in the E12.5 *Gli3* mutant despite some differences in the dissected tissue and in the effects of the mutations on Gli3. Whereas *Inpp5e* mutants showed an increased formation of neurons in the dorsolateral telencephalon, E11.5 *Gli3* conditional mutants initially presented with delayed neurogenesis in the rostromedial dorsal telencephalon which resolved by E12.5. This discrepancy likely stems from variations in analyzed tissues and reflects the lateral to medial neurogenic gradient in the developing cortex. Notably, negative regulators of neurogenesis such as *Ptn* and *Mycn* were downregulated in *Inpp5e* embryos but upregulated in E11.5 *Gli3* conditional mutants (Table S1). Furthermore, unlike *Inpp5e* mutants where Gli3R levels decreased by approximately 50% but remained present in significant amounts (Hasenpusch et al. 2020), *Gli3* conditional embryos harbored a loss-of-function mutation in the cortex by about E11.5 (Hasenpusch et al. 2018). This contrast can explain the varying degrees to which *Pappa* and *Sall3* expression are affected by the 2 mutations. Despite these differences, both mutants appear to converge on similar gene expression changes at E12.5 with a remarkable 95% of DEGs to be regulated in the same direction. This strong convergence elucidates the successful rescue of the *Inpp5e* cortical malformations through Gli3R restoration and provides a unique foundation for identifying genes downstream of cilia and of Gli3 during corticogenesis.

### *Pappa* and *Sall3* are direct targets for ciliary signaling and Gli3

To confirm whether our candidate list encompasses genes directly regulated by Gli3, we focussed on 2 specific examples: *Sall3* and *Pappa*. These genes may have important roles in patterning or regulating cortical growth/neurogenesis aligning with established *Gli3* and *Inpp5e* phenotypes and the relevant terms identified in our GO analysis. We demonstrated that Gli3 can indeed bind in vivo and in vitro to evolutionarily conserved sites within regulatory elements of both genes. Notably, mutating these binding sites led to ectopic activation of reporter genes in the dorsal telencephalon. Collectively, these findings establish *Pappa* and *Sall3* as novel direct targets of Gli3, highlighting Gli3’s role as a transcriptional repressor in suppressing their expression in the developing cortex.

The role of these 2 genes in the developing cortex remain to be fully understood, yet their known functions offer intriguing possibilities. Previous studies have shown complex interactions between *Sall* genes and the *Gli3*/*Shh* pathway and placed these genes upstream (Kawakami et al. 2009) and downstream of Shh signaling (Kawakami et al. 2009; Nishi et al. 2015) or revealed cooperative interactions (Akiyama et al. 2015). *Sall* gene function in neural development is only poorly understood and is complicated by complex and overlapping expression patterns (Fig. S2; Ott et al. 2001; Sato et al. 2003; Bohm et al. 2008; Harrison et al. 2012) suggesting potential redundant functions as seen during limb development and neural tube closure (Bohm et al. 2008; Kawakami et al. 2009). *SALL3* deletion has been implicated in 18q23 deletion syndrome (Kohlhase et al. 1999), characterized by intellectual disability and limb abnormalities. In mice, loss of *Sall3* resulted in palate deficiency, abnormalities in cranial nerves, and perinatal lethality (Parrish et al. 2004). While telencephalic development was not explored in this mutant, ectopic *Sall3* expression in the cortical primordium is known to interfere with the nuclear transport of the Sall1 transcription factor (Sweetman et al. 2003), while cytoplasmic retention of a truncated Sall1 protein disrupts cilia formation and function (Bozal-Basterra et al. 2018). Moreover, reduced *Sall1* function might lead to premature neuronal differentiation and increased neuron formation as observed in *Sall1* global and conditional mutants (Harrison et al. 2012).

*Pappa* encodes a secreted protein that has not been located to primary cilia in several studies of the ciliary proteome (Mick et al. 2015; Boldt et al. 2016; Aslanyan et al. 2023). It plays a critical role in Igf signaling by proteolytically cleaving Igf binding proteins (Igfbps), thereby releasing sequestered Igfs for signaling (Lawrence et al. 1999). These secreted factors, their receptors and Igfbps are expressed in the developing cortex and surrounding tissue (Fig. S3; Ayer-le Lievre et al. 1991; Bondy et al. 1992; Higginbotham et al. 2013) suggesting that cortical RGCs are responsive to Igfs. This notion is supported by our finding that the increased *Pappa* expression in *Inpp5e* mutants correlates with enhanced Akt signaling which is activated upon Igf binding to its receptor (Fig. S4). Moreover, interfering with Igf signaling reduced brain growth, while Igf2 from the cerebrospinal fluid stimulated neural progenitor proliferation (Beck et al. 1995; Kappeler et al. 2008; Liu et al. 2009; Lehtinen et al. 2011). Hence, *Pappa*’s widespread upregulation likely contributes to increased proliferation in E11.5 *Gli3* conditional mutants. In contrast, the restricted ectopic *Pappa* expression in *Inpp5e* mutants coincided with an increase in direct neurogenesis. Interestingly, Igf signaling can promote neuronal differentiation under certain conditions. *Nestin*/*Igf1* transgenic mice showed a preferential increase in the formation of layer V neurons (Hodge et al. 2005) and Igf2 also promoted adult neural stem cell differentiation through upregulation of *Cdkn1c* (Lozano et al. 2023) which is augmented in *Inpp5e* mutants but decreased in E11.5 *Gli3* conditional mutants (Table S1). Thus, the effects of Igf signaling on neural progenitor behavior appear developmentally regulated and require further investigations.

In conclusion, our findings address a gap in our knowledge of genes acting downstream of cilia in corticogenesis and provide a detailed list of candidate target genes highlighting 2 novel potential pathways for further exploration. Thereby, we shed light on the mechanisms by which cilia orchestrate aspects of cortical development and contribute to a more comprehensive apprehension of ciliary functions.

## Supplementary Material

Supplementary_Figures_Cerebral_Cortex_bhae480

Supplementary_Table1_bhae480

Supplementary_Tables_2_to_4_bhae480
