# Supplementary material for: Identification of Pappa and Sall3 as Gli3 direct target genes acting downstream of cilia signaling in corticogenesis
Source: Cereb Cortex. 2024 Dec 24;34(12):bhae480. doi: 10.1093/cercor/bhae480 (PMC11666469; doi:10.1093/cercor/bhae480)
Supplement: Supplementary_Figures_Cerebral_Cortex_bhae480 [file supplementary_figures_cerebral_cortex_bhae480.docx]

**Supplementary Figures**

**Supplementary Figure 1**


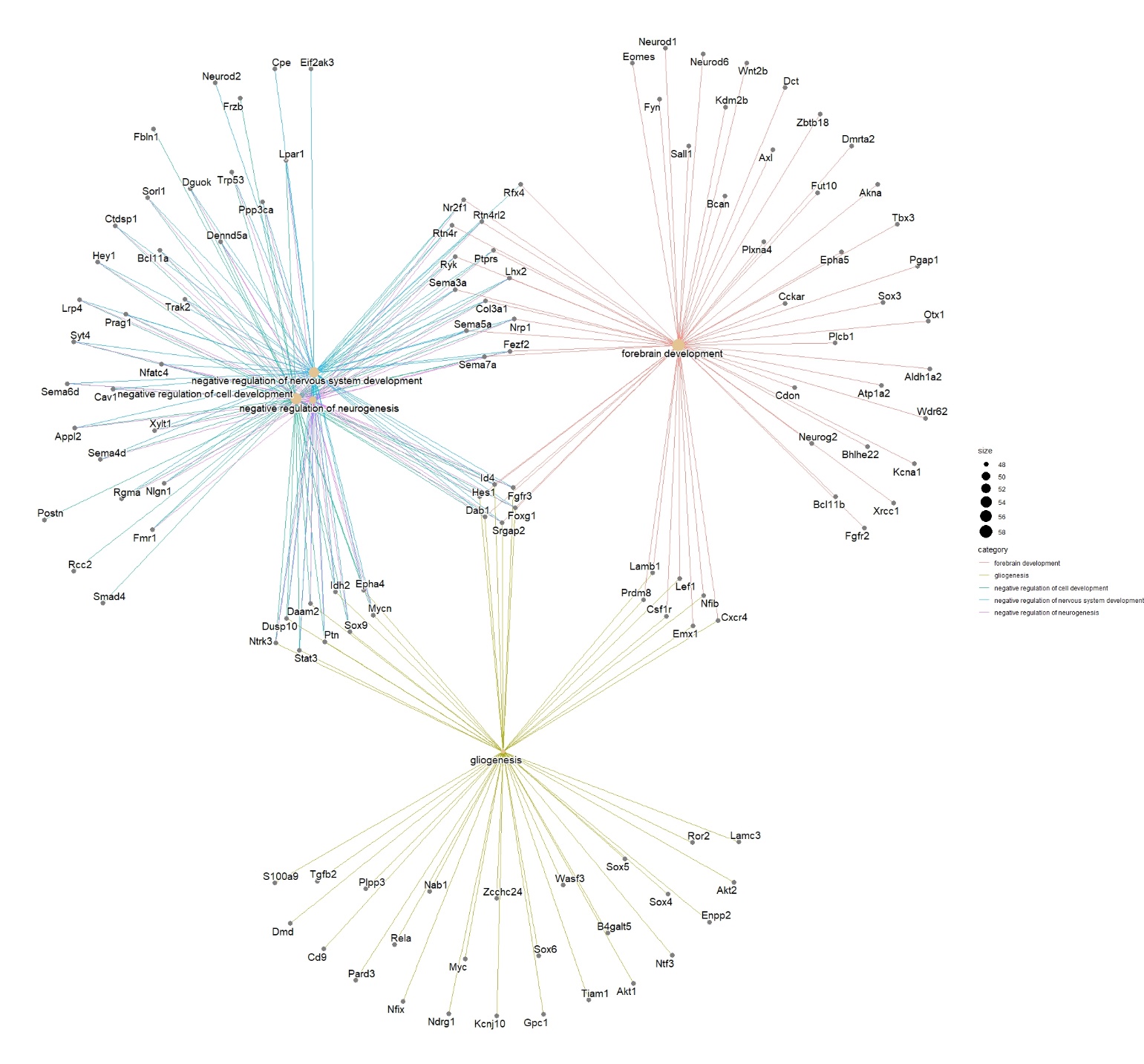


**Supplementary Figure 1: Network plot of down-regulated genes**.

**Supplementary Figure 2**


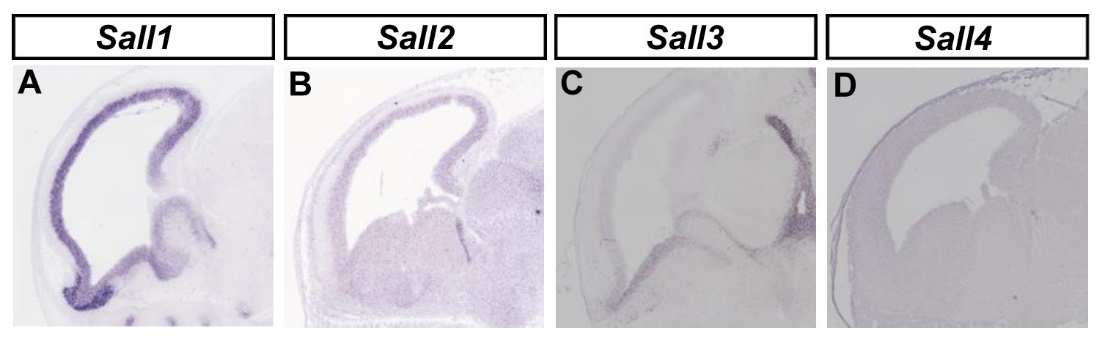


**Supplementary Figure 2: *Sall* gene expression in the E14.5 mouse cortex**. Images were taken from Genepaint.

**Supplementary Figure 3**


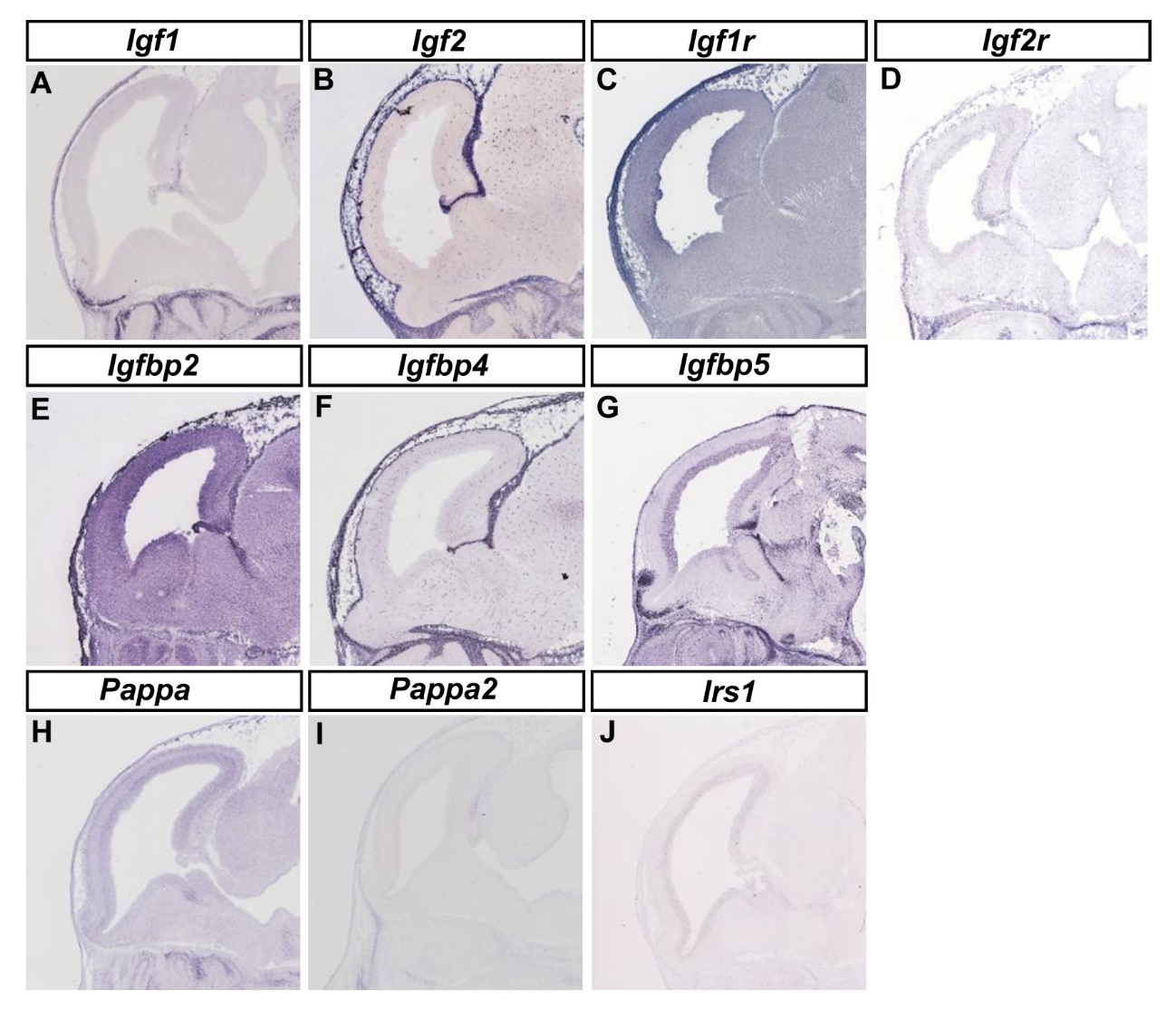


**Supplementary Figure 3: Expression of genes encoding Igf signalling components in the E14.5 mouse cortex**. Images were taken from Genepaint.

**Supplementary Figure 4**


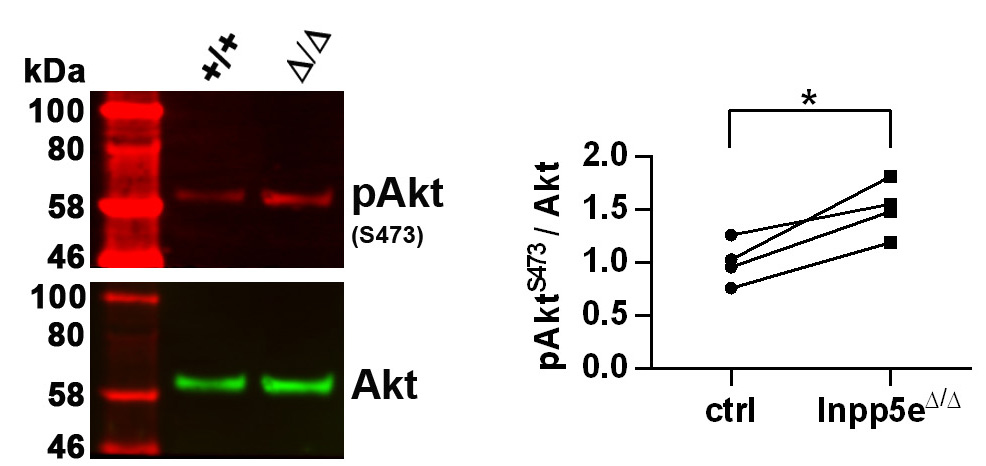


**Supplementary Figure 4: Akt signalling in *Inpp5e*^Δ/Δ^ embryos.** Western blot analyses on dorsal telencephalic tissue from E12.5 embryos with the indicated antibodies. The pAkt^S473^/total Akt ratio is increased in E12.5 *Inpp5e*^Δ/Δ^ embryos. For quantification, a paired t-test was used to evaluate expression levels of the phosphorylated versus unphosphorylated proteins in four control/*Inpp5e*^Δ/Δ^ embryo pairs derived from four different litters. Statistical data are presented as means ± 95% confidence intervals (CI); n = 4; * p < 0.05.
