# Supplementary material for: Identification of Pappa and Sall3 as Gli3 direct target genes acting downstream of cilia signaling in corticogenesis
Source: Cereb Cortex. 2024 Dec 24;34(12):bhae480. doi: 10.1093/cercor/bhae480 (PMC11666469; doi:10.1093/cercor/bhae480)
Supplement: Supplementary_Tables_2_to_4_bhae480 [file supplementary_tables_2_to_4_bhae480.docx]

**Supplementary Table 2**

**Cloning *Pappa* enhancer**

Forward primer AAAGGTACCTCTCTGCTACCATCCGTCCT

Reverse primer AAAGTCGACAGGCCTGCAGAAGATAAGGG

**Cloning *Sall3* enhancer**

Forward primer AAAAAGCTTGCATCTCAAGTCGGACGAGG

Reverse primer AAACTCGAGATAGCGACCGTTCTGCACTT

**Supplementary Table 3**

**Oligonucleotides used for mutagenesis**

*Pappa* binding site 1

Forward primer GCCATCTCGGGGGT**AT**CCCTCCGCTCTTTC

Reverse primer GAAAGAGCGGAGGG**AT**ACCCCCGAGATGGC

*Pappa* binding site 2

Forward primer GAGCCATAGATGAGGGT**AT**CCATTTGGGGGAGG

Reverse primer CCTCCCCCAAATGG**AT**ACCCTCATCTATGGCTC

*Sall3* binding site 1

Forward primer GTTCTGGGGCGC**AT**ACCCAGGTCGGGAAGC

Reverse primer GCTTCCCGACCTGGGT**AT**GCGCCCCAGAAC

*Sall3* binding site 2

Forward primer GGGGACCTCAGGGT**AT**CCCAGAGTTCTGGG

Reverse primer CCCAGAACTCTGGG**AT**ACCCTGAGGTCCCC

**Supplementary Table 4**

**Oligonucleotides used for EMSA**

*Pappa* Binding site 1

Biotinylated forward primer Btn-CATCTCGGGGGTGGCCCTC

Forward primer CATCTCGGGGGTGGCCCTC

Reverse primer GAGGGCCACCCCCGAGATG

Forward mutated primer CATCTCGGGGGT**AT**CCCTC

Reverse mutated primer GAGGG**AT**ACCCCCGAGATG

*Pappa* Binding site 2

Biotinylated forward primer Btn-CATAGATGAGGGTGGCCATTTG

Forward primer CATAGATGAGGGTGGCCATTTG

Reverse primer CAAATGGCCACCCTCATCTATG

Forward mutated primer CATAGATGAGGGT**AT**CCATTTG

Reverse mutated primer CAAATGG**AT**ACCCTCATCTATG

*Sall3* Binding site 1

Biotinylated reverse primer CGACCTGGGTGGGCGCC-Btn

Forward primer GGCGCCCACCCAGGTCG

Reverse primer CGACCTGGGTGGGCGCC

Forward mutated primer GGCGC**TA**ACCCAGGTCG

Reverse mutated primer CGACCTGGGT**TA**GCGCC

*Sall3* Binding site 2

Biotinylated reverse primer CTCTGGGCCACCCTGAGGTC-Btn

Forward primer GACCTCAGGGTGGCCCAGAG

Reverse primer CTCTGGGCCACCCTGAGGTC

Forward mutated primer GACCTCAGGGT**AT**CCCAGAG

Reverse mutated primer CTCTGGG**AT**ACCCTGAGGTC
